# Lifestyle Changes for the Treatment of Hypertension

**DOI:** 10.1007/s11906-026-01401-7

**Published:** 2026-11-14

**Authors:** Jacob Stein, Anthony Thompson, Angeli Thomas, Arun Janakiraman, Jordana B. Cohen

**Affiliations:** 1 Perelman School of Medicine, University of Pennsylvania, Philadelphia, PA USA; 2 Penn State College of Medicine, Pennsylvania State University, Hershey, PA USA; 3 Washington DC Veterans Affairs Medical Center, DC Washington, USA

**Keywords:** Hypertension, Potassium-based salt substitute, Exercise, Weight loss, Diet, Alcohol

## Abstract

**Purpose of Review:**

Lifestyle interventions are foundational to hypertension prevention and treatment. We review recent evidence for weight management, dietary patterns, sodium and potassium intake, physical activity, stress reduction, alcohol reduction, and avoidance of blood pressure-raising substances, with emphasis on implementation and contemporary guidelines.

**Recent Findings:**

Recent trials extend Dietary Approaches to Stop Hypertension (DASH) benefits to grocery delivery in populations facing barriers to food access, while potassium-enriched salt-substitution studies support cardiovascular benefit but require attention to hyperkalemia risk. Alcohol reduction lowers blood pressure most among individuals with higher baseline intake or blood pressure, and avoidance of other pressor substances remains important. Evidence for time-restricted eating and stress-reduction strategies is uncertain.

**Summary:**

Lifestyle modification is recommended for all adults with elevated blood pressure or hypertension, but sustained adherence and equitable access are major barriers. Future research should prioritize scalable, durable, culturally responsive strategies and outcomes beyond short-term blood pressure change.

## Introduction

Hypertension is a major public health issue that affects more than 1.4 billion adults aged 30–79 years worldwide [1]. Lifestyle modification is recommended for all adults with elevated blood pressure (BP) or hypertension. Current guidelines recommend antihypertensive medication in addition to lifestyle modification for adults with BP ≥ 140/90 mm Hg and for selected adults with stage 1 hypertension at increased cardiovascular risk; among lower-risk adults, medication is recommended if BP remains ≥ 130/80 mm Hg after 3–6 months of lifestyle modification [2]. Multiple modifiable lifestyle and dietary factors influence BP and the risk of developing hypertension. These include achieving or maintaining a healthy weight, managing stress, following a Dietary Approaches to Stop Hypertension (DASH)-style eating pattern, reducing dietary sodium, increasing dietary potassium, engaging in regular aerobic, resistance, and isometric exercise, and reducing or eliminating alcohol intake and other substances that can increase BP. The feasibility and effectiveness of these strategies are also shaped by social and structural factors, including access to affordable healthy foods, neighborhood food environments, and the availability of sustained behavioral and dietary support. These factors may contribute to disparities in both implementation and long-term BP control.

**Fig. 1:**
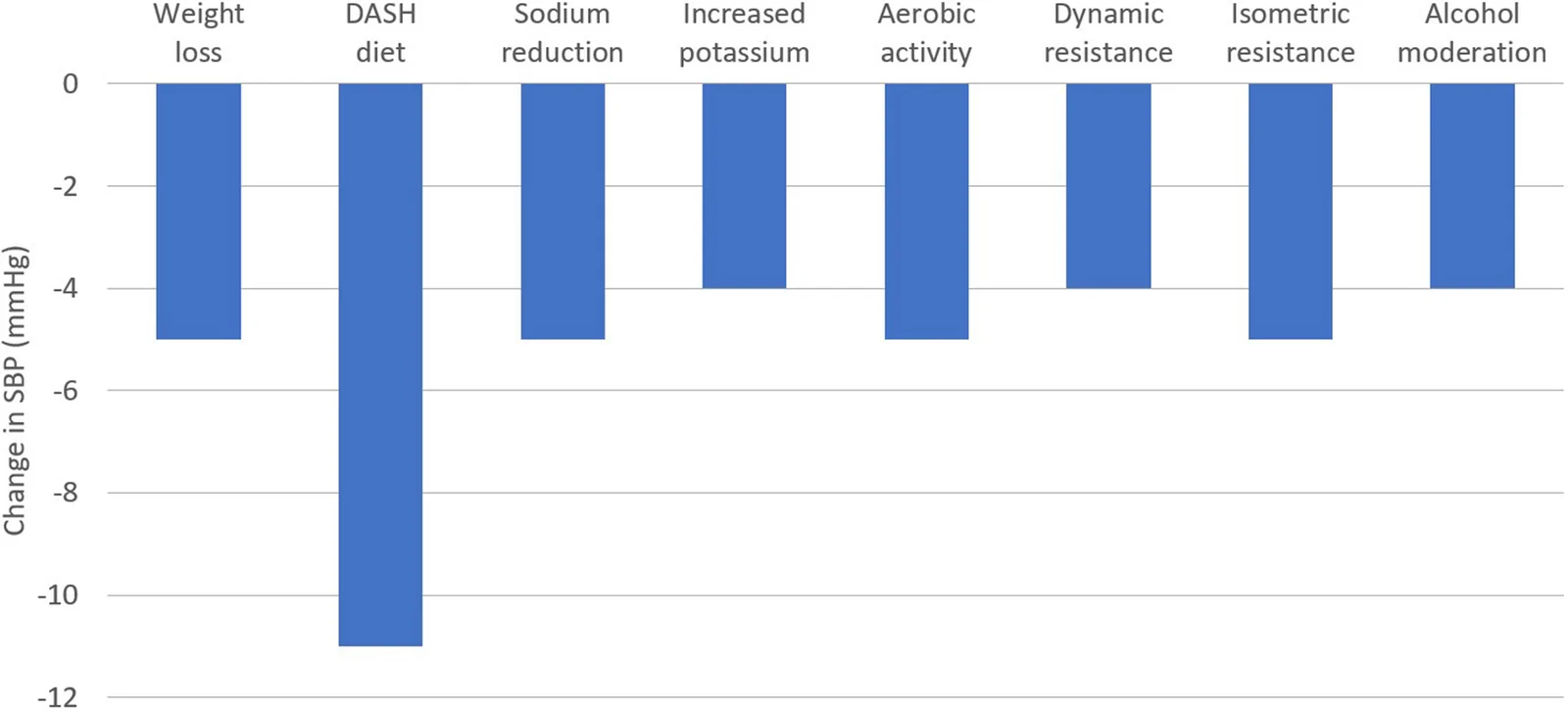
Approximate impact of lifestyle modifications on systolic blood pressure among adults with hypertension. This figure represents the average anticipated SBP reduction from individual lifestyle interventions, and is adapted, with permission, from Table 15 of the 2017 American College of Cardiology/American Heart Association Blood Pressure Guideline [33]. Effects are approximate average estimates and should not be summed across interventions

The approximate impact of individual lifestyle modifications on systolic BP (SBP) is shown in the Fig. 1. Implementation considerations and important caveats for these interventions are summarized in the Table 1. Adopting a combination of lifestyle measures can produce complementary BP reductions, although long-term adherence is often challenging.

**Table 1:** Implementation considerations for lifestyle interventions for hypertension

Intervention	Approach	Implementation Considerations	Important Caveats
Weight management	Aim for sustained ≥ 5% body-weight reduction when overweight or obesity is present	Caloric restriction and physical activity can be combined	Maintenance rather than initial weight loss is often the major challenge; BP benefit depends on sustained weight reduction
Physical activity	Aim for ≥ 150 min/week of moderate aerobic activity plus resistance exercise on ≥ 2 days/week	Multiple forms are effective; structured programs may improve adherence; can be individualized to functional status, comorbidities, access, and patient preference; even lower-intensity activity that interrupts sedentary time may lower BP	Isometric exercise evidence is promising but based heavily on office BP and relatively short trials
Stress reduction	Meditation, breathing control, or yoga may be used as adjuncts	Low cost and preference-sensitive	BP evidence is predominantly short term and low/very-low certainty
DASH diet	Diet rich in fruits, vegetables, whole grains, and low-fat dairy; limits saturated and total fat	Most effective in combination with sodium reduction	Limited by food access and affordability
Sodium reduction	< 2,300 mg/day, ideally < 1,500 mg/day	Most US sodium is added before purchase	Food reformulation and policy may be as important as individual counseling; very low sodium targets may be difficult to sustain
Potassium intake	3,500-5,000 mg/day, preferably from food	Potassium-rich dietary patterns overlap substantially with DASH diet	Individualize based on serum potassium, kidney function, and medications affecting potassium excretion
Potassium-enriched salt substitutes	Replace cooking/table salt with a potassium-enriched salt substitute (typically ~ 25%-30% KCl)	Particularly attractive where cooking/table salt accounts for substantial sodium intake	Less applicable where processed foods dominate sodium intake; monitor potassium or consider avoiding in those with high hyperkalemia risk
Alcohol	Abstinence is the optimal goal; if alcohol is continued, ≤ 1 drink/day for women and ≤ 2/day for men	Largest BP reductions occur among people with higher baseline intake and BP	Direct evidence that alcohol abstinence reduces cardiovascular events is lacking; many guidelines recommend limiting intake rather than abstinence
Caffeine	< 300 mg/day	Limit caffeine further with severe uncontrolled hypertension	Acute caffeine exposure and habitual coffee consumption have different BP effects
Illicit drugs	Avoid illicit sympathomimetics, including cocaine and methamphetamine	Specifically ask, because patients may not spontaneously report use	Acute effects are well-established; longitudinal risks are less clear, although greater lifetime exposure has been associated with elevated BP
Supplements	Avoid products with known pressor effects, including ephedra, bitter orange/ p-synephrine, yohimbine, and glycyrrhizic acid	Specifically ask, because patients may not spontaneously report use	Formulations vary, potentially requiring careful review of individual ingredients
Abbreviations: *BP* blood pressure, *DASH *Dietary Approaches to Stop Hypertension			

## Weight Loss with Diet and Exercise

### Obesity and Hypertension Risk

Excess adiposity is strongly associated with higher BP and the development of hypertension. Higher body mass index (BMI), weight gain, and increasing waist circumference have each been associated with greater risk for incident hypertension [3–5]. Obesity is increasing in prevalence worldwide. According to the World Health Organization, in 2022, 43% of adults were overweight and 16% were living with obesity [6]. Multiple mechanisms likely contribute to obesity-associated hypertension, including renal sodium retention, activation of the sympathetic nervous system and renin-angiotensin-aldosterone system, insulin resistance, inflammation, oxidative stress, and vascular dysfunction [5, 7, 8]. In the Framingham Heart Study, overweight or obesity was estimated to account for approximately 26% of new hypertension cases in men and 28% in women [9]. Accordingly, weight loss achieved through dietary modification, increased physical activity, or both is an important component of lifestyle management for the prevention and treatment of hypertension [5]. The American Heart Association (AHA)/American College of Cardiology (ACC) hypertension guideline estimates a ~ 1 mm Hg reduction in SBP for each 1 kg reduction in body weight [2]. Dietary and exercise interventions can promote weight loss while also lowering BP through mechanisms that are at least partly independent of changes in body weight.

### Dietary Modifications for Weight Loss and Hypertension

Caloric restriction is an established approach to weight loss and can meaningfully reduce BP among adults with hypertension [5, 10]. In a meta-analysis of randomized controlled clinical trials (RCTs) evaluating weight-reducing diets in individuals with hypertension, dietary interventions produced a mean weight reduction of approximately 4 kg and a corresponding mean BP reduction of 4.5/3.2 mm Hg [11]. Nonetheless, long-term maintenance of diet-induced weight loss is challenging, and sustained BP benefit is likely dependent on maintaining the associated weight reduction [5].

More recently, time-restricted eating has been proposed as an alternative strategy to facilitate weight loss and possibly BP reduction [12]. However, in a 12-week randomized isocaloric feeding trial among adults with obesity, weight loss was similar with time-restricted eating and a usual eating pattern, suggesting that weight loss observed with time-restricted eating in other studies may largely reflect reductions in caloric intake [13]. Whether meal timing itself promotes weight loss or BP reduction independent of reduced caloric intake remains uncertain.

### Exercise for the Management of Hypertension

Regular physical activity is associated with lower risk of developing hypertension, and structured exercise lowers BP among individuals with established hypertension [14]. Aerobic, dynamic resistance, and isometric exercise can each lower BP. Aerobic exercise has been studied most extensively [15]. In a systematic review and dose-response meta-analysis of 34 RCTs including 1,787 adults with hypertension, the dose-response meta-analysis estimated 1.8/1.2 mm Hg lower SBP/diastolic BP (DBP) for each additional 30 min per week of aerobic exercise, respectively, with the largest estimated reductions observed at approximately 150 min per week [16]. These effects are partly independent of weight loss, supporting a direct role for physical activity in hypertension management rather than solely as a strategy for weight reduction [5].

Dynamic resistance and isometric exercise also lower BP. A meta-analysis of 24 RCTs among older adults with prehypertension or hypertension found that resistance training reduced SBP and DBP by approximately 6.9 and 3.4 mm Hg, respectively [17]. Similarly, a meta-analysis of 12 RCTs among adults with hypertension found that isometric resistance training reduced office BP by approximately 7.5/3.2 mm Hg, although reductions in mean 24-hour ambulatory BP were not observed [18]. In the Exercise and Nutrition interventions for CardiOvasculaR hEalth (ENCORE) trial, a multicomponent intervention combining the DASH diet, caloric restriction, and exercise produced greater BP reduction compared to the DASH diet alone (-16.1/-9.9 mm Hg vs. -11.2/-7.5 mm Hg) [19]. Collectively, these findings support incorporating aerobic and resistance-based exercise according to patient preferences, abilities, and comorbidities.

## Stress Reduction and Mind-Body Approaches

Stress-reduction interventions have been evaluated as adjunctive strategies for BP lowering, although the evidence is less robust than for dietary and exercise interventions. The 2025 AHA/ACC guideline states that transcendental meditation, breathing-control techniques, and yoga may be reasonable adjuncts for prevention or treatment of elevated BP and hypertension [2]. Short-term trials suggest modest BP reductions with several approaches; the guideline cites mean reductions of approximately 5/2 mm Hg with transcendental meditation and 5/3 mm Hg with breathing-control interventions, while evidence for yoga is less consistent. However, these estimates are derived largely from small, heterogeneous, short-duration studies, and the durability of BP lowering remains uncertain. Consistent with these limitations, a 2025 systematic review and network meta-analysis of 182 studies found that although several relaxation approaches were associated with lower BP over short-term follow-up, certainty of evidence was very low because of risk of bias, potential publication bias, imprecision, and limited long-term follow-up [20]. Thus, stress-reduction and mind-body interventions may be considered according to patient preference, but should complement rather than replace lifestyle interventions with stronger evidence or indicated pharmacologic therapy.

## Dietary Approaches to Lower Blood Pressure

### Sodium Restriction

Reducing dietary sodium is a well-established strategy for lowering BP. The 2025 AHA/ACC hypertension guideline recommends a dietary sodium intake of < 2,300 mg/day, with an ideal limit of < 1,500 mg/day [2]. The BP response to sodium reduction is dose-dependent and varies according to baseline characteristics and the duration of the intervention. In a meta-analysis of 133 RCTs including 12,197 adults in which sodium intake was assessed using 24-hour urinary sodium excretion, Huang et al. found that a mean reduction in sodium excretion of 130 mmol/day lowered SBP and DBP by 4.3 and 2.1 mm Hg, respectively [21]. In multivariable meta-regression, each 50 mmol/day reduction in urinary sodium excretion (which equates to about 1,150 mg of dietary sodium) was associated with 1.1 mm Hg greater reduction in SBP and 0.3 mm Hg greater reduction in DBP. For the same degree of sodium reduction, BP lowering was greater among individuals with higher baseline BP and older adults. Very short trials also appeared to underestimate the effect: among trials lasting < 15 days, each 50 mmol/day reduction in sodium excretion lowered SBP by 1.1 mm Hg, compared with 2.1 mm Hg in trials of longer duration. However, there was little evidence that progressively longer intervention duration conferred additional BP lowering beyond this, and relatively few trials extended beyond 6 months [21].

Achieving substantial sodium reduction through individual behavior change can be difficult because much of dietary sodium is incorporated into foods before they reach the consumer. In a US study using repeated dietary recalls and direct assessment of salt added during food preparation and at the table, approximately 71% of sodium intake was attributable to sodium added outside the home during food manufacturing, processing, or commercial preparation, whereas only approximately 11% was added during home food preparation or at the ​table [22]. Meaningful reductions in sodium intake may require policy measures that promote or mandate changes in food manufacturing, processing, and commercial preparation, in addition to individual strategies such as choosing lower-sodium foods and limiting discretionary salt use. A recent example comes from France, where national targets were introduced to progressively reduce salt content across commonly consumed breads. A 2026 population-based modeling study estimated that full implementation would reduce average salt intake by 0.35 g/day (~ 140 mg/day sodium) and mean SBP by only 0.2 mm Hg at the individual level; when applied across the population, this modest shift was estimated to avert approximately 8,400 hospitalizations and 1,200 deaths annually [23].

### The DASH Diet

The DASH diet lowers BP independently of sodium restriction and weight loss. The DASH diet emphasizes fruits, vegetables, whole grains, and low-fat dairy products while limiting saturated and total fat. In the original RCT of 459 adults with or without hypertension, sodium intake and body weight were held constant. Compared with a control diet typical of US intake, the DASH diet reduced BP by 5.5/3.0 mm Hg overall and by 11.4/5.5 mm Hg among participants with hypertension [24]. Across a broader evidence base, a meta-analysis of 30 RCTs including 5,545 participants found that DASH reduced SBP and DBP by approximately 3.2 and 2.5 mm Hg, respectively, compared with control diets [25].

### Sodium Restriction Combined with the DASH Diet

Sodium reduction and the DASH diet have complementary BP-lowering effects. In the controlled-feeding DASH-Sodium trial, 412 adults with elevated BP or stage 1 hypertension who were not taking antihypertensive medications were randomized to the DASH or control diet; within their assigned dietary pattern, participants were also assigned to consume high-, intermediate-, and low-sodium diets in random order. Both DASH and sodium reduction lowered BP, with the greatest reductions observed when the interventions were combined. Compared with a high-sodium control diet, the low-sodium DASH diet reduced SBP by 7.1 mm Hg among participants without hypertension and 11.5 mm Hg among those with hypertension [26]. Secondary analyses suggested different time courses for the two interventions: most of the BP reduction attributable to DASH occurred within the first week, whereas the effect of sodium reduction continued to increase over the 4-week feeding period [27]. Additionally, greater BP reductions with combined DASH and sodium restriction were observed among participants with higher baseline BP [28].

The efficacy of the DASH diet and sodium restriction was largely established in controlled feeding studies, but recent trials have evaluated strategies to translate these interventions into real-world settings. In the GoFresh trial, 180 Black adults with elevated BP or untreated hypertension living in communities with limited grocery-store access were randomized to 12 weeks of home-delivered, low-sodium DASH-patterned groceries with dietitian counseling or monetary stipends for self-directed grocery shopping. The intervention reduced SBP by 3.4 mm Hg and DBP by 2.4 mm Hg more than the self-directed approach at 3 months, although the BP benefit was no longer evident 3 months after grocery delivery ended [29]. The subsequent GoFreshRx trial tested a similar intervention among 176 Black adults receiving antihypertensive treatment and demonstrated a 5.0 mm Hg greater reduction in SBP and 1.8 mm Hg greater reduction in DBP at 3 months compared with self-directed grocery shopping; BP benefits were partially maintained 3 months after the intervention ended [30]. These findings demonstrate that DASH- and sodium-focused dietary interventions can lower BP outside of controlled feeding settings, while also highlighting the importance of sustained access to healthy foods and dietary support for maintaining benefit.

### Potassium Intake

Increasing potassium intake can lower BP, particularly among individuals with hypertension and those consuming higher-sodium diets. The 2025 AHA/ACC hypertension guideline recommends a potassium intake of 3,500-5,000 mg/day, ideally from potassium-rich foods, for adults with elevated BP or hypertension; moderate potassium supplementation may also be considered when appropriate [2]. In a meta-analysis of 22 RCTs including 1,606 adults, increased potassium intake reduced BP by 3.5/2.0 mm Hg overall and by 5.3/3.1 mm Hg among participants with hypertension, with little effect among participants without hypertension [31]. The BP-lowering effect also appeared greater in populations with higher baseline sodium intake. A subsequent dose-response meta-analysis of 32 RCTs found a nonlinear relationship between potassium supplementation and BP, with maximal BP lowering at moderate increases in potassium intake and attenuation of benefit at higher doses [32]. Consequently, current guidelines favor increasing potassium primarily through dietary sources [2]. Notably, in individuals with chronic kidney disease (CKD) or taking medications that impair potassium excretion, the 2025 AHA/ACC guideline recommends monitoring serum potassium with increased potassium intake. This represents a shift from earlier guidance that treated CKD itself as a potential contraindication to potassium supplementation [33]. Recent American Society of Nephrology Kidney Health Guidance similarly does not recommend routine restriction of dietary potassium solely on the basis of CKD and instead emphasizes individualized assessment of hyperkalemia risk [34]. Given the high prevalence of hypertension among individuals with CKD, potassium intake should be individualized according to serum potassium, medication use, and other risk factors for hyperkalemia.

### Potassium-enriched Salt Substitution

Potassium-enriched salt substitutes provide a complementary strategy by simultaneously reducing sodium intake and increasing potassium intake. In a stepped-wedge cluster-randomized trial of 2,376 adults from six villages in Peru, replacing household salt with a formulation containing 75% sodium chloride and 25% potassium chloride reduced BP by 1.3/0.8 mm Hg; among participants without hypertension at baseline, the intervention was associated with a 51% lower risk of developing hypertension [35]. The strongest cardiovascular outcome evidence comes from the Salt Substitute and Stroke Study (SSaSS), an open-label cluster-randomized trial of 20,995 high-risk adults from 600 rural Chinese villages. Over a mean 4.7 years, use of the same 75% sodium chloride/25% potassium chloride formulation lowered SBP by 3.3 mm Hg and reduced stroke, major cardiovascular events, and all-cause mortality by 14%, 13%, and 12%, respectively, compared with regular salt [36]. Serious adverse events attributed to hyperkalemia did not differ significantly between groups, although serum potassium was not systematically monitored. A network meta-analysis of 34 RCTs similarly found that potassium-enriched salt substitutes containing 25–40% potassium chloride and 60–79% sodium chloride lowered SBP by approximately 4–5 mm Hg compared with regular salt, although evidence for cardiovascular and mortality benefit is largely driven by SSaSS [37].

Because most salt-substitution trials have been conducted in populations in which discretionary salt contributes substantially to sodium intake, their applicability may be more limited in settings such as the US, where a substantial proportion of dietary sodium is incorporated into commercially prepared foods [22]. Nonetheless, the 2025 AHA/ACC guideline states that potassium-based salt substitutes can be useful for prevention and treatment of elevated BP and hypertension, particularly when discretionary salt used in home food preparation or seasoning is a major source of sodium intake [2]. Similarly, the World Health Organization released a guideline in 2025 that now conditionally recommends replacing regular table salt with potassium-containing lower-sodium salt substitutes when table salt is used [38]. As with potassium intake more broadly, potassium-enriched salt substitutes warrant additional monitoring in individuals with CKD or taking medications that impair potassium excretion and should generally be avoided in those at high risk for hyperkalemia.

## Reduction or Avoidance of Blood Pressure-raising Substances

### Alcohol

Alcohol consumption is associated with higher BP and greater risk of developing hypertension in a dose-dependent manner. Longitudinal evidence suggests that this relationship extends to relatively low levels of alcohol intake. In a dose-response meta-analysis of seven nonexperimental cohort studies including 19,548 adults, alcohol intake was associated with progressively greater increases in BP over a median follow-up of 5.3 years, without evidence of a threshold for the association with SBP. Compared with no alcohol intake, consumption of 12 and 48 g/day of alcohol was associated with SBP that was 1.3 and 4.9 mm Hg higher, respectively [39]. Reducing alcohol consumption lowers BP, with larger effects among individuals with higher baseline alcohol intake and higher baseline BP [40, 41]. In a meta-analysis of 36 trials including 2,865 participants, reducing alcohol intake was associated with progressively greater BP reduction among those consuming more than 2 drinks/day at baseline. Among participants consuming ≥ 6 drinks/day, reducing alcohol intake by approximately 50% lowered BP by 5.5/4.0 mm Hg; no significant BP reduction was demonstrated among those consuming ≤ 2 drinks/day [40]. Observational evidence suggests that smaller benefits may also occur with cessation at lower levels of intake. For example, in a longitudinal observational analysis of nearly 59,000 adults undergoing repeated health examinations in Japan, each 10-g/day decrease in alcohol consumption was associated with reductions in SBP and DBP of approximately 0.9 and 0.8 mm Hg, respectively, with dose-dependent BP reductions after alcohol cessation and corresponding increases after alcohol initiation [42]. Accordingly, the 2025 AHA/ACC hypertension guideline now recommends abstinence as the optimal goal for adults who consume alcohol; for those who continue to drink, intake should be reduced to no more than 1 drink/day for women or 2 drinks/day for men [2].

### Caffeine

Caffeine can acutely raise BP, although its acute effects differ from those of habitual coffee consumption. In a systematic review and meta-analysis of controlled trials among individuals with hypertension, ingestion of 200–300 mg of caffeine increased SBP and DBP by 8.1 and 5.7 mm Hg, respectively; the increase occurred within the first hour and persisted for at least 3 h [43]. In contrast, the same review found no sustained increase in BP in the limited trials evaluating coffee consumption over 2 weeks, and cohort studies did not demonstrate greater cardiovascular risk with habitual coffee consumption. A later meta-analysis of eight RCTs evaluating caffeine supplementation in adults found smaller but significant increases in SBP and DBP of 1.9 and 1.7 mm Hg, respectively, with larger effects at higher caffeine doses [44]. Given the acute pressor effects of caffeine and more limited evidence for adverse effects with habitual coffee consumption, the 2025 AHA/ACC guideline recommends limiting caffeine intake to < 300 mg/day and avoiding more than 1 cup of coffee daily in individuals with severe uncontrolled hypertension [2].

### Illicit Drugs

The 2025 AHA/ACC hypertension guideline recommends discontinuation or avoidance of recreational drugs that increase BP, including cocaine, methamphetamine, and synthetic cathinones (“bath salts”) [2]. Illicit sympathomimetic drugs, particularly cocaine and methamphetamine, can acutely raise BP through increased sympathetic activity and vasoconstriction and can precipitate severe hypertension and cardiovascular complications [45, 46]. Evidence linking repeated illicit stimulant use to sustained chronic hypertension is less consistent. In a study of 301 individuals hospitalized for treatment of cocaine dependence, 20% had acutely elevated BP that normalized within 1 day, whereas the prevalence of sustained hypertension was not greater than that observed in a population-based comparison group [47]. In a subsequent cross-sectional analysis of 5,861 US adults, lifetime cocaine use was independently associated with greater odds of BP ≥ 130/85 mm Hg, with a stronger association among individuals reporting greater lifetime exposure to cocaine; methamphetamine and heroin use were not independently associated with elevated BP [48]. Despite the lack of an association with chronic hypertension in that analysis, methamphetamine is well established to cause acute BP elevation and substantial cardiovascular toxicity [46].

### Herbal and Dietary Supplements

Several herbal and dietary supplements can increase BP and their use should be specifically assessed when evaluating individuals with hypertension. The 2025 AHA/ACC hypertension guideline recommends avoiding herbal supplements with known pressor effects, including ephedra (Ma Huang) and yohimbine, and separately recommends avoiding black licorice [2]. Ephedra contains sympathomimetic alkaloids, principally ephedrine, and was removed from the US dietary supplement market in 2004 because of cardiovascular and other safety concerns [49]. Bitter orange (*Citrus aurantium*), which contains the sympathomimetic alkaloid p-synephrine and has been marketed in weight loss supplements as an alternative to ephedra, may also increase BP. In a systematic review and meta-analysis of 18 placebo-controlled trials, longer-term BP effects were evaluable in two trials including 75 participants; after approximately 8 weeks, p-synephrine-containing products increased SBP and DBP by 6.4 and 4.3 mm Hg, respectively, without evidence of meaningful weight loss [50]. Yohimbine, an α2-adrenergic receptor antagonist found in some supplements marketed for weight loss or sexual enhancement, increases sympathetic outflow and has also been shown to raise BP [51].

Licorice represents a mechanistically distinct cause of supplement- or food-related BP elevation. Glycyrrhizic acid in licorice is metabolized to glycyrrhetinic acid, which inhibits 11β-hydroxysteroid dehydrogenase type 2 and thereby permits cortisol-mediated activation of the mineralocorticoid receptor, resulting in a mineralocorticoid excess phenotype characterized by sodium retention, potassium loss, suppressed renin and aldosterone, and increased BP. In a meta-analysis of eight RCTs including 541 participants, interventions containing glycyrrhizic acid increased SBP and DBP by 3.5 and 1.3 mm Hg, respectively, whereas licorice preparations dominated by flavonoids without substantial glycyrrhizic acid did not significantly affect BP [52]. Because supplement formulations vary substantially and patients may not spontaneously report their use, clinicians should specifically ask about herbal products and dietary supplements in individuals with elevated or difficult-to-control BP.

## Conclusions and Future Directions

Lifestyle modifications can produce clinically meaningful and complementary reductions in BP and are central to hypertension prevention and treatment. The strongest evidence supports achieving or maintaining a healthy weight, a DASH-style dietary pattern, sodium reduction, adequate dietary potassium or potassium-enriched salt substitution in appropriate patients, regular aerobic, resistance, and isometric exercise, and reduction or elimination of alcohol and other pressor substances. The principal challenge is less whether these approaches work than how to make them durable and accessible. Recent pragmatic trials of grocery delivery and community-level salt substitution illustrate the potential of implementation strategies that reduce reliance on sustained individual behavior change. These studies also highlight how limited food access and the loss of sustained dietary support can constrain the implementation and durability of effective lifestyle interventions, with important implications for disparities in BP control. Future studies should prioritize long-term adherence, cardiovascular and kidney outcomes, equitable implementation across diverse settings, and safety in populations at risk for hyperkalemia. Additional studies are also needed to clarify whether time-restricted eating has BP effects independent of caloric restriction and whether stress-reduction interventions produce sustained BP lowering. Lifestyle interventions should complement, rather than delay, antihypertensive medication when pharmacologic therapy is indicated.

## Key References

Papers of particular interest, published recently, have been highlighted:

- ​Jones DW, Ferdinand KC, Taler SJ, Johnson HM, Shimbo D, Abdalla M, et al. 2025 AHA/ACC/AANP/AAPA/ABC/ACCP/ACPM/AGS/AMA/ASPC/NMA/PCNA/SGIM Guideline for the Prevention, Detection, Evaluation and Management of High Blood Pressure in Adults: A Report of the American College of Cardiology/American Heart Association Joint Committee on Clinical Practice
  - ○Provides the current US guideline framework for lifestyle management of hypertension, including updated recommendations for sodium, potassium, alcohol, caffeine, exercise, and stress reduction.
- Webster KE, Halicka M, Bowater RJ, Parkhouse T, Stanescu D, Punniyakotty AV, et al. Effectiveness of stress management and relaxation interventions for management of hypertension and prehypertension: systematic review and network meta-analysis. BMJ Med. 2025;4(1):e001098.
  - ○Provides a contemporary synthesis of stress-reduction interventions, showing possible short-term BP benefit but very low certainty of evidence and limited long-term data.
- Juraschek SP, Col H, Ferro K, Turkson-Ocran RN, Cluett JL, Davis RB, et al. DASH-patterned groceries and effects on blood pressure in adults treated for hypertension: the GoFreshRx randomized trial. Nat Med. 2026;32(6):2128-36
  - ○Demonstrates that home-delivered DASH-patterned groceries with dietary support can meaningfully and sustainably lower BP in adults receiving antihypertensive treatment, extending DASH efficacy into a pragmatic real-world setting.
- Biruete A, Anderson C, Bernier-Jean A, Clase CM, Clegg D, Crews DC, et al. ASN Kidney Health Guidance on Potassium and Phosphorus Food Additives. Journal of the American Society of Nephrology : JASN. 2025;36(11):2244-58.
  - ○Updates the approach to potassium intake in patients with CKD, emphasizing individualized hyperkalemia risk rather than routine potassium restriction solely because CKD is present.
- Lai H, Nesrallah G, Guyatt GH, Vernooij RWM, Liu J, Zhao W, et al. Comparative effects of salt substitutes on blood pressure, cardiovascular events and mortality: a systematic review and network meta-analysis. BMC Med. 2026;24(1):106.
  - ○Provides contemporary evidence that potassium-enriched salt substitutes lower BP, while clarifying that cardiovascular outcome evidence is driven largely by SSaSS.

## Data Availability

No datasets were generated or analysed during the current study.
